# Gesundheit wohnungsloser Menschen während der COVID-19-Pandemie

**DOI:** 10.1007/s00103-023-03739-8

**Published:** 2023-07-19

**Authors:** Victoria van Rüth, André Hajek, Fabian Heinrich, Benjamin Ondruschka, Klaus Püschel, Franziska Bertram

**Affiliations:** 1 Institut für Rechtsmedizin, Universitätsklinikum Hamburg-Eppendorf, Butenfeld 34, 22529 Hamburg, Deutschland; 2 Institut für Gesundheitsökonomie und Versorgungsforschung, Universitätsklinikum Hamburg-Eppendorf, Hamburg, Deutschland Martinistr. 52, 20246

**Keywords:** Obdachlos, SARS-CoV‑2, COVID-19-Pandemie, Gesundheit, Versorgungszustand, Houseless, SARS-CoV‑2, COVID-19 pandemic, Health, Supply situation

## Abstract

Die Lebenssituation und die Gesundheit wohnungsloser Menschen unterscheiden sich in vielerlei Hinsicht von denen der Allgemeinbevölkerung. Die Vermutung liegt nahe, dass es sich bei wohnungslosen Menschen um eine besonders vulnerable Personengruppe während der Coronavirus-Disease-2019(COVID-19)-Pandemie handelt. In dieser narrativen Übersichtsarbeit soll die aktuelle Literatur zur Gesundheit und Versorgung von wohnungslosen Menschen während der COVID-19-Pandemie zusammengefasst werden. Recherchiert wurde zwischen Dezember 2022 und Februar 2023. Neben der aktuellen nationalen und internationalen Literatur sollen insbesondere die Ergebnisse des „National survey on psychiatric and somatic health of homeless individuals“ (NAPSHI-Studie) synoptisch dargestellt werden; diese untersucht psychische und somatische Erkrankungen sowie die Versorgung wohnungsloser Menschen in Deutschland.

Wohnungslose Menschen sind häufig psychisch und somatisch erkrankt und haben einen eingeschränkten Zugang zum medizinischen Regelsystem. Versorgungseinrichtungen mit Gruppenräumen und Schlafsälen stellen ein Risiko für ein Ausbruchsgeschehen in der COVID-19-Pandemie dar. Wie vermutet, zeigten sich im Verlauf der Pandemie bei wohnungslosen Menschen häufiger als in der Allgemeinbevölkerung Hinweise für Severe Acute Respiratory Syndrome Coronavirus Type 2(SARS-CoV‑2)-Infektionen, viele davon schienen allerdings asymptomatisch zu verlaufen. Eine hohe Rate an unwissentlich infizierten wohnungslosen Menschen könnte zur Verbreitung der Viruserkrankung beigetragen haben. Trotzdem war ein unkontrolliertes COVID-19-Ausbruchsgeschehen, vor dem einige Wissenschaftler:innen zu Beginn der Pandemie warnten, nicht zu beobachten.

## Abstract (en)

The living situation and health of homeless people differs from the general population in many ways. It is reasonable to assume that the homeless population has been particularly vulnerable during the coronavirus disease 2019 (COVID-19) pandemic. This narrative review will summarize the current literature on the health and care of homeless people during the COVID-19 pandemic. The literature research was performed between December 2022 and February 2023. In addition to the current national and international literature, findings from the “National Survey on the Psychiatric and Somatic Health of Homeless Individuals” (NAPSHI study) will be synopsized, examining psychiatric and somatic diseases as well as the care for homeless people in Germany.

Homeless individuals are often mentally and physically ill and have limited access to the regular medical care system. Facilities with group rooms and dormitories pose a risk for outbreaks during the COVID-19 pandemic. As suspected, evidence of Severe Acute Respiratory Syndrome Coronavirus Type 2 (SARS-CoV‑2) infections emerged more frequently in homeless individuals than in the general population during the pandemic. Many of the infected individuals were asymptomatic. High rates of those unknowingly infected homeless individuals may have contributed to the spread of the viral disease. However, uncontrolled COVID-19 outbreaks, as feared by some researchers at the beginning of the pandemic, were not observed.

Im Dezember 2019 traten erste Infektionen mit Severe Acute Respiratory Syndrome Coronavirus Type 2 (SARS-CoV‑2) in China auf; rasant breitete sich das Virus über alle Kontinente und durch sämtliche Gesellschaftsschichten aus. Bereits zu Beginn der Coronavirus-Disease-2019(COVID-19)-Pandemie postulierten die Autoren Tsai und Wilson eine besonders hohe Vulnerabilität wohnungsloser Menschen in den USA, bedingt durch 2 Faktoren: Wohnungslose Menschen hätten ein erhöhtes Risiko für Infektionserkrankungen durch ihre Umwelt- und Lebensbedingungen, wie die Übernachtung in Sammelunterkünften und einen erschwerten Zugang zu medizinischer Versorgung. Außerdem hätten wohnungslose Menschen durch viele Vorerkrankungen ein erhöhtes Risiko für einen schweren Verlauf von Infektionserkrankungen [1].

Diesem Kommentar folgten wissenschaftliche Untersuchungen zur Gesundheit und Versorgung wohnungsloser Menschen an unterschiedlichen Standorten. Es ist jedoch zu beachten, dass Studien, die während der COVID-19-Pandemie durchgeführt wurden, nur eingeschränkt miteinander vergleichbar sind. Grund dafür sind sich stetig verändernde Regulationen und fluktuierende Infektionszahlen im Verlauf der Pandemie.

Diese Arbeit soll eine Übersicht über aktuell verfügbare Daten zu den von Tsai und Wilson aufgeworfenen Fragestellungen liefern und die bisher generierte Evidenz zu Prävalenz und Prävention von SARS-CoV‑2-Infektionen unter wohnungslosen Menschen zusammentragen. Dabei wird insbesondere über den zwischen Juni und September 2021 durchgeführten, multizentrischen „National survey on psychiatric and somatic health of homeless individuals“ (NAPSHI-Studie) aus Deutschland berichtet und diese Erkenntnisse in die nationale und internationale Literatur eingebettet. Insgesamt konnten in die NAPSHI-Studie 651 Wohnungslose in den Metropolregionen um Hamburg, Leipzig, Frankfurt und München eingeschlossen werden. Weitere hier dargestellte Arbeiten wurden zwischen Dezember 2022 und Februar 2023 in der Datenbank „Pubmed“ recherchiert, zusätzlich wurden Veröffentlichungen der Bundesarbeitsgemeinschaft Wohnungslosenhilfe e. V. (BAG W) und des Robert Koch-Instituts (RKI) berücksichtigt.

## Hohes Risiko für eine SARS-CoV‑2-Infektion durch Umwelt- und Lebensbedingungen

### Soziodemografische Daten wohnungsloser Menschen in Deutschland

In Deutschland leben etwa 263.000 wohnungslose Menschen (2022), davon sind etwa 68 % männlich, 31 % weiblich und 1 % divers [2, 3]. 178.000 von ihnen kommen regelmäßig in Notunterkünften der Kommunen unter, etwa 37.400 Menschen leben dauerhaft auf der Straße [2, 4]. Etwa 40 % der wohnungslosen Menschen nutzen Versorgungssysteme nicht, weil sie es als zu gefährlich erachten oder dort mit zu vielen anderen Personen untergebracht seien [2, 5]. Dies führt dazu, dass die Ergebnisse aus Studien mit wohnungslosen Menschen selten generalisierbar sind, da nur bestimmte Gruppen innerhalb dieser Kohorte erreicht werden können.

Daher ist es schwierig, die genaue Anzahl und die Lebensbedingungen wohnungsloser Menschen in Deutschland zu erfassen. Aufgrund abweichender Schätzungsmodelle von BAG W und dem statistischen Bundesamt sind auch die Zahlen wohnungsloser Menschen im Zeitverlauf eingeschränkt vergleichbar; dennoch ist ein deutlicher Anstieg zwischen den Jahren 2008 und 2016 zu erkennen [6, 7].

Im Rahmen der NAPSHI-Studie gaben 46 % der Studienteilnehmenden eine abgeschlossene Schulbildung an, 67 % waren ledig. Im Median waren die Befragten 18 Monate ohne festen Wohnsitz [2, 5].

### Versorgung und Unterbringung wohnungsloser Menschen

In Deutschland ist die Versorgungslandschaft wohnungsloser Menschen heterogen strukturiert, unterliegt aber der auf dem Ordnungsrecht fußenden Unterbringung und Notversorgung. Diese ist durch ein ausdifferenziertes Hilfesystem von Städten und Gemeinden sicherzustellen [8]. Darüber hinaus gibt es zuwendungs- oder spendenfinanzierte Träger, die teils individuell, teils deutschlandweit agieren, wie beispielsweise Diakonie oder Caritas [2]. Vielfältig sind auch die Versorgungsangebote: Einige Einrichtungen bieten Übernachtungen mit oder ohne Mahlzeiten an, andere sind Tagesaufenthaltsstätten. Wieder andere Einrichtungen bieten medizinische oder sozialpädagogische Beratung, supervidierten Drogenkonsum oder Kleidung an. Entsprechend dem Angebot der jeweiligen Einrichtung verfügen diese über unterschiedlich qualifiziertes Personal, häufig ergänzt durch ehrenamtliche Mitarbeiter:innen [9].

Trotz dieses Engagements ergab eine Befragung bei Trägern der Wohnungsnotfallhilfe bereits vor der COVID-19-Pandemie (2019), dass die untersuchten Einrichtungen nicht immer ausreichende Versorgungs- und Präventionsangebote bieten und die Infrastruktur zur Etablierung solcher häufig fehle [9].

Durch die COVID-19-Pandemie wurde die Versorgungssituation wohnungsloser Menschen in Deutschland weiter verschärft. Bestrebungen, die Belegungsdichten zu reduzieren, und die deutschlandweite Schließung von Einrichtungen verursachten eine schlagartige Reduktion der Versorgungsangebote [10]. Eine bundesweite Umfrage der BAG W im Mai 2020 verdeutlicht die Besorgnis um die Existenz der Einrichtungen und den Ausblick, dass Engpässe bei der Versorgung Wohnungsloser mit Lebensmitteln und bei der medizinischen Versorgung auftreten könnten [11]. Eine Großzahl der Einrichtungen hatte keine Möglichkeiten zur Isolation oder Behandlung erkrankter Personen, weiterhin konnten keine ausreichenden Hygienemaßnahmen, Kontaktverfolgung und SARS-CoV‑2-Testungen angeboten werden [2, 10]. Dies stellte vor allem zu Beginn der Pandemie im Jahr 2020 die Versorgung wohnungsloser Menschen in Deutschland vor große Hürden [10].

Um diesen Schwierigkeiten gezielt zu begegnen, wurde von der BAG W im Oktober 2020 ein 10-Punkte-Plan veröffentlicht, der die Erkenntnisse des ersten halben Jahres der COVID-19-Pandemie darstellte und die Aufrechterhaltung der Versorgung ermöglichen sollte. Zu diesem Zeitpunkt konnten 95 % der Unterkünfte eine Pandemie-adaptierte Versorgung anbieten [12].

### Zugang zu und Inanspruchnahme von medizinischen Leistungen

Während der Allgemeinbevölkerung im Erkrankungsfall verschiedene ambulante und stationäre medizinische Hilfen zur Verfügung stehen, ist dies für wohnungslose Menschen nur selten der Fall. Grund dafür ist vermutlich häufig ein fehlender Krankenversicherungsschutz. In der NAPSHI-Studie gaben nur 67,7 % der Studienteilnehmenden an, krankenversichert zu sein [5]. In der in Berlin durchgeführten POINT-Studie, einer Erhebung des RKI zu übertragbaren Erkrankungen unter wohnungslosen Menschen im Jahr 2021, waren nur 43 % aller Befragten versichert [13]. Hinzu kommt, dass durch Sprachbarrieren oder Stigmatisierungsangst sowie durch Misstrauen gegenüber den Behandelnden häufig nur Notfälle medizinisch versorgt werden, während chronische oder infektiologische Erkrankungen gar nicht oder erst zu spät behandelt werden [14, 15].

In der NAPSHI-Studie im Jahr 2021 gaben 72,0 % der Studienteilnehmenden an, in den letzten 12 Monaten ambulanten Arztkontakt gehabt zu haben. Im Median gaben die wohnungslosen Menschen mit Arztkontakt 3 ambulante Konsultationen pro Jahr an. Außerdem berichteten 42,4 %, im letzten Jahr stationär im Krankenhaus behandelt worden zu sein. Dabei betrug die mediane Dauer 6 Nächte und die mediane Anzahl der Krankenhausaufenthalte 4 jährlich [5].

In einer monozentrischen Studie aus Hamburg zwischen Mai und Juni 2020 gaben etwa 65 % der wohnungslosen Menschen an, keine medizinische Leistung in Anspruch genommen zu haben, weil sie keinen Bedarf hierfür sahen. Obwohl hier 34 % der Befragten nicht krankenversichert waren, spielte dies nach eigener Einschätzung der wohnungslosen Menschen keine Rolle für die Nutzung medizinischer Hilfen [16].

Es ist anzunehmen, dass fehlender Zugang zum medizinischen Regelsystem, wo chronische Erkrankungen behandelt werden und Aufklärung über Infektionen und Impfungen stattfinden kann, sowie eingeschränkte Möglichkeiten zur adäquaten Infektionsprophylaxe in den versorgenden Einrichtungen das Risiko für eine SARS-CoV‑2-Infektion für wohnungslose Menschen im Vergleich zur Allgemeinbevölkerung erhöhen.

Im Rahmen von COVID-19-Impfaktionen für wohnungslose Menschen konnte gezeigt werden, dass diese erfolgreicher verlaufen, wenn sie speziell auf die Lebenssituation von Menschen im Wohnungsnotfall angepasst sind [17, 18]. Der dauerhafte Ausbau ebensolcher niedrigschwelliger Angebote könnte die medizinische Versorgung wohnungsloser Menschen nachhaltig verbessern.

## Hohes Risiko für einen schweren Verlauf durch hohe Prävalenzen psychischer und somatischer Erkrankungen

### Somatische Gesundheit

Im Vergleich zur Allgemeinbevölkerung ist die Lebenserwartung von wohnungslosen Menschen reduziert, bei Männern um 21,6 und bei Frauen um 17,4 Jahre [19]. Eine Studie aus Frankreich aus dem Jahr 2021 zeigt ein mittleres Sterbealter wohnungsloser Menschen von 40,9 Jahren [20]. In der deutschen Allgemeinbevölkerung liegt die Lebenserwartung etwa doppelt so hoch [21].

Alkohol- oder Drogenmissbrauch, Rauchen, Übergewicht und Mangelernährung sind bekannte Risikofaktoren für viele chronische somatische Erkrankungen. Eine Übersichtsarbeit über Studien vor der COVID-19-Pandemie zeigte, dass diese Risikofaktoren häufiger bei wohnungslosen Menschen als in der Allgemeinbevölkerung vorkommen [22].

Früh in der COVID-19-Pandemie wurde beschrieben, dass besonders Herz-Kreislauf- und Stoffwechselerkrankungen Risikofaktoren für das Versterben an einer SARS-CoV‑2-Infektion darstellen [23]. Systematische Übersichtsarbeiten aus 2017 und 2020 zeigen, dass Herz-Kreislauf-Erkrankungen bei 7–37 % der untersuchten wohnungslosen Menschen, Lungenerkrankungen bei 6–24 % und muskuloskelettale Erkrankungen bei mehr als 20 % diagnostiziert wurden. Verletzungen und Intoxikationen sind bei 5–15 % und infektiöse oder parasitäre Erkrankungen bei 10–16 % der Population beschrieben [14, 22].

In der NAPSHI-Studie lag die Prävalenz kardiovaskulärer Risikofaktoren deutlich höher: Es zeigte sich ein möglicher Bluthochdruck bei 38,5 % der Teilnehmenden, ein Diabetes mellitus bei 4,4 % und eine Erhöhung der Blutfette bei 17,6 % [5]. Zusätzlich untersuchte die NAPSHI-Studie die Häufigkeit ärztlich diagnostizierter chronischer Erkrankungen bei den Studienteilnehmenden. Häufige ärztliche Diagnosen betrafen das Herz-Kreislauf-System, die Leber und die Lunge. Bei etwa der Hälfte der Befragten ergab sich ein Hinweis auf das mögliche Vorliegen von nicht diagnostizierten somatischen Erkrankungen [5].

Unabhängig von der COVID-19-Pandemie sind hohe Prävalenzen verschiedener infektiologischer Erkrankungen unter wohnungslosen Menschen in Deutschland beschrieben. Die NAPSHI-Studie zeigt eine erhöhte Prävalenz von Infektionen mit dem humanen Immunschwäche-Virus (HIV) im Vergleich zur Allgemeinbevölkerung [5]: Diese lag unter Wohnungslosen bei 0,7 % (4/651); in der POINT-Studie wurde eine Prävalenz von 2,8 % (6/213) gezeigt. Passend dazu berichteten die Studienteilnehmenden eine häufige Exposition gegenüber Risikofaktoren: 71 % der Teilnehmenden gaben Haftaufenthalte, 48 % Tätowierungen aus nicht professionellen Settings an [13]. Weiterhin berichteten 74 % der Teilnehmenden, in den letzten 12 Monaten ungeschützten Sexualkontakt gehabt zu haben [24, 25].

Zusätzlich wurden in der POINT-Studie die Prävalenzen von Infektionserkrankungen sowie Impftiter für Hepatitis B, C (HBV/HCV) und Tuberkulose untersucht. Hier zeigte sich eine aktive HBV-Infektion bei 1,9 %, eine abgelaufene Infektion bei 17 %, geimpft waren 26 % der Studienteilnehmenden. 24 % zeigten Hinweise auf aktiven oder früheren HCV-Kontakt und 16 % eine aktive HCV-Infektion [13]. Im Vergleich dazu konnte im „Hamburg survey of homeless individuals“ aus dem Jahr 2020 eine Hepatitis-A-Impfung oder -Infektion bei 40 % der Studienteilnehmenden nachgewiesen werden, eine Infektion mit Hepatitis E bei 29 %, Impfung oder Infektion mit HBV bei 27 % und eine durchgemachte HCV-Infektion bei 10 % [26]. Klinisch zeigte sich in der POINT-Studie kein Verdachtsfall für eine Tuberkulose, der Interferon-y-Test war jedoch bei 14,4 % der Teilnehmenden positiv als Hinweis auf eine latente Tuberkulose-Infektion [13].

### Psychische Gesundheit

Trotz hoher Prävalenzen somatischer Erkrankungen unter wohnungslosen Menschen zeigt eine Untersuchung von Nielsen und Kolleg:innen aus dem Jahr 2011, dass vor allem psychische Erkrankungen, insbesondere das Vorliegen einer substanzbezogenen Störung, mit früherem Versterben wohnungsloser Menschen assoziiert war [20]. Auch in der COVID-19-Pandemie waren psychischen Erkrankungen mit einem erhöhten Risiko für das Auftreten einer SARS-CoV‑2-Infektion assoziiert und im Falle einer Infektion mit einer erhöhten Mortalität [27, 28].

Es ist zu beachten, dass wohnungslose Menschen bereits vor der COVID-19-Pandemie unter einer reduzierten psychischen Gesundheit litten: Systematische Übersichtsarbeiten beschreiben einen Alkohol- oder Drogenabusus bei bis zu 36,7 % beziehungsweise 21,7 % der wohnungslosen Menschen, gefolgt von Schizophrenien (12,6 %) und Depressionen (12,6 %; [29]). Es zeigte sich, dass wohnungslose Menschen, die im Krankenhaus behandelt werden, signifikant häufiger Suizidgedanken hatten als Personen aus der Allgemeinbevölkerung (19,4 % vs. 2,9 %; [30]). Es wird daher zu regelmäßigen, strukturierten Screening-Untersuchungen der psychischen Gesundheit bei wohnungslosen Menschen geraten [29, 30]. Betroffene sollten an niederschwellige Beratungsstellen, sozialpsychiatrische Dienste und Therapeut:innen angebunden werden, die auf die Situation der Wohnungslosigkeit spezialisiert sind, um langfristig eine Verbesserung des psychischen Gesundheitszustandes erzielen zu können.

Während der COVID-19-Pandemie gaben 42,3 % der Teilnehmenden der NAPSHI-Studie einen kritischen Alkoholkonsum an [5]. Weitere Studien konnten zeigen, dass sich bei etwa der Hälfte der wohnungslosen Menschen, die in Befragungen keinen chronisch kritischen Alkoholkonsum angaben, dennoch in einer toxikologisch-chemischen Untersuchung Hinweise auf einen solchen ergaben [31]. Es ist daher anzunehmen, dass die Prävalenzen des chronisch kritischen Alkoholkonsums deutlich höher liegen als hier durch Fragebögen ermittelt. Weiterhin berichteten 29,4 % der Teilnehmenden der NAPSHI-Studie vom regelmäßigen Konsum illegaler Substanzen [5].

In der Allgemeinbevölkerung zeigte sich im Verlauf der COVID-19-Pandemie eine deutliche Zunahme psychischer Erkrankungen [32, 33]. In Untersuchungen zwischen März und Mai 2020 (Anfänge der COVID-19-Pandemie) aus Spanien hatten 63 % der befragten wohnungslosen Menschen psychische Erkrankungen [34]. Longitudinale Daten zur psychischen Gesundheit wohnungsloser Menschen im Verlauf der COVID-19-Pandemie gibt es derzeit nicht. Jedoch wurden sowohl im „Hamburg survey of homeless individuals“ im Jahr 2020 und in der NAPSHI-Studie im Jahr 2021 psychische Erkrankungen anhand validierter Screening-Fragebögen untersucht. So ergaben sich zu Beginn der COVID-19-Pandemie (2020) deutlich erhöhte Punktprävalenzen für sowohl generalisierte Angststörungen als auch Depressionen im Kollektiv wohnungsloser Menschen im Vergleich zur Allgemeinbevölkerung mit 19,7 % und 22,5 % [35].

Im Kontrast dazu steht, dass wohnungslose Menschen nur in 23,6 % der Fälle davon berichten, dass eine psychische Erkrankung ärztlich diagnostiziert wurde. Die NAPSHI-Studie ergab daher bei 70 % der Befragten Hinweise auf eine mögliche nichtdiagnostizierte psychische Erkrankung [5]. Auch wohnungslose Menschen selbst geben in Studien an, dass der wesentliche Grund für das Fortbestehen der Wohnungslosigkeit ihr psychischer Gesundheitszustand sei [5, 36].

Trotz methodischer Limitationen, wie der erschwerten Erfassung von Langzeitdaten, Selektionsbias und dem erschwerten Zugang zu wohnungslosen Menschen, zeichnen aktuelle Studien an mehreren Standorten das Bild einer deutlich erhöhten Prävalenz psychischer und somatischer Erkrankungen bei wohnungslosen Menschen im Vergleich zur Allgemeinbevölkerung. Es werden insbesondere erhöhte Prävalenzen somatischer Erkrankungen, die mit einem schweren COVID-19-Verlauf assoziiert sind, beschrieben. Das Vorliegen von psychischen Erkrankungen kann zur erschwerten Prävention und Therapie einer SARS-CoV‑2-Infektion beitragen [27]. Interessanterweise schätzen wohnungslose Menschen 2020 dennoch ihre gesundheitsbezogene Lebensqualität subjektiv besser ein als die deutsche Allgemeinbevölkerung [37]. Mögliche Gründe könnten sein, dass wohnungslose Menschen geringere Ansprüche an ihre gesundheitsbezogene Lebensqualität haben oder sich an ihre Umstände hinreichend angepasst fühlen [37, 38].

## Infektionen und Immunität unter wohnungslosen Menschen während der COVID‑19-Pandemie

Sehr früh zu Beginn der COVID-19-Pandemie untersuchten Forscher:innen in den USA die Ausbreitung von SARS-CoV‑2 in Wohnungslosenunterkünften mittels Polymerase-Ketten-Reaktion-Testung (PCR). Sie zeigten im April 2020 bei 11,7 % der Studienteilnehmenden Hinweise für eine akute oder kürzlich abgelaufene Infektion mit SARS-CoV‑2 [39]. Bei einer maximalen Inzidenz von 0,4 % in der amerikanischen Allgemeinbevölkerung in diesem Zeitraum kann hier von einem Ausbruch und Infektionsspot gesprochen werden [40].

Auch eine Arbeitsgruppe in Frankreich untersuchte die Häufigkeit von SARS-CoV‑2-Infektionen bei wohnungslosen Menschen. Zwischen Juni und August 2020 wurden in 48 unterschiedlichen Versorgungseinrichtungen in Marseille Antikörper gegen SARS-CoV‑2 im Blut bestimmt. Hier ergab sich eine Seroprävalenz von 5,6 %. Die Studie zeigte außerdem, dass wohnungslose Menschen, die in Notunterkünften wohnen, häufiger Infektionen durchmachten als solche, die auf der Straße leben. Dies lässt vermuten, dass das Bewohnen von Sammelunterkünften für wohnungslose Menschen ein Infektionsrisiko darstellt, trotz vermeintlich besserer Möglichkeiten zur Einhaltung von Hygienemaßnahmen. Die Infektionshäufigkeit für wohnungslose Menschen war außerdem gegenüber der französischen Allgemeinbevölkerung erhöht [41].

In Belgien lag die Prävalenz von SARS-CoV‑2 ermittelt über PCR-Testung im gleichen Zeitraum (Sommer 2020) unter wohnungslosen Menschen bei 4,9 % [42].

Etwa ein Jahr später, zwischen Juli und September 2021, untersuchte die NAPSHI-Studie das SARS-CoV‑2-Infektionsgeschehen unter wohnungslosen Menschen in Deutschland. Hier wiesen etwa 18 % der Kohorte Antikörper entsprechend einer durchgemachten SARS-CoV‑2-Infektion auf. Im Vergleich zur deutschen Allgemeinbevölkerung war dies deutlich erhöht: In Rheinhessen wurde der Anteil an 25- bis 88-Jährigen mit durchgemachter SARS-CoV‑2-Infektion bis Juni 2021 anhand von Antikörper- und PCR-Tests auf 4,9 % geschätzt, in München konnten bis Oktober 2021 bei 7,9 % der Studienteilnehmenden Antikörper gegen SARS-CoV‑2 auf eine Infektion zurückgeführt werden [43, 44].

Ähnliche Verhältnisse zeigt auch eine aktuelle Studie aus Frankreich auf, in welche 1249 wohnungslose Menschen zwischen August und Dezember 2020 in Marseille eingeschlossen wurden. Mit einem dreimonatigen Abstand wurden SARS-CoV‑2-Antikörpertests durchgeführt. Zum ersten Zeitpunkt betrug die Seroprävalenz unter wohnungslosen Menschen 6,0 % und stieg innerhalb der 3 Monate auf 18,9 %, verglichen mit 3,0 % respektive 6,5 % in der Allgemeinbevölkerung. Auch hier hatten Menschen, die regelmäßig in Notunterkünften schliefen, ein signifikant erhöhtes Risiko für eine SARS-CoV‑2-Infektion [45].

In einer Übersichtsarbeit zu SARS-CoV‑2-Infektionen bei wohnungslosen Menschen wurden Studien analysiert, die zwischen April und Juli 2020 durchgeführt wurden. Hier zeigte sich, dass bei PCR-Testungen in Ausbruchssituationen bis zu 14 % der Tests positiv waren, die Gesamtprävalenz über den ganzen Erhebungszeitraum lag allerdings nur bei 2 %. Eine systematische Analyse konnte für übergreifende Zeiträume zwischen Dezember 2019 und März 2021 eine durchschnittliche COVID-19-Prävalenz von 2,3 % unter wohnungslosen Menschen und in Ausbruchsgeschehnissen von bis zu 32 % detektieren. In solchen Situationen waren zusätzlich bis zu 15 % der Mitarbeiter:innen ebenfalls mit SARS-CoV‑2 infiziert [46].

Unter wohnungslosen Menschen zeigen sich also zu verschiedenen Zeitpunkten und an verschiedenen Orten erhöhte Prävalenzen von SARS-CoV‑2-Infektionen im Vergleich zur Allgemeinbevölkerung. Bei Zusammenschau dieser internationalen Studien darf nicht vergessen werden, dass sich kulturelle, soziale und Versorgungssystem-bedingte Unterschiede ergeben. Zur verlässlichen Einschätzung der Situation in Deutschland und zur Entwicklung und Bewertung geeigneter Interventionen bedarf es weiterer Daten aus Deutschland.

Interessanterweise zeichnet sich studienübergreifend unter wohnungslosen Menschen eine deutliche Diskrepanz zwischen dem Symptomerleben und einer Infektion mit SARS-CoV‑2 ab. In Dänemark gaben seropositive und seronegative wohnungslose Menschen vergleichbar häufig an, SARS-CoV‑2-assoziierte Symptome wahrgenommen zu haben [47]. Auch eine Untersuchung aus Kanada 2020 konnte keine Assoziation zwischen SARS-CoV‑2-Positivität und Symptomen zeigen [48]. Dazu passend zeigte sich in einer Screening-Untersuchung 2020 in Belgien, dass 93 % der positiv getesteten wohnungslosen Menschen asymptomatisch blieben [42]. Auch in der NAPSHI-Studie gaben 80 % jener wohnungslosen Menschen, die Antikörper für eine durchgemachte SARS-CoV‑2-Infektion aufwiesen, an, bisher keine Infektion wissentlich durchgemacht zu haben. In den Ergebnissen der Gutenberg COVID-19-Studie, welche in der Allgemeinbevölkerung in Rheinland-Pfalz zwischen 2020 und 2022 durchgeführt wurde, verliefen lediglich 42,3 % aller Infektionen unwissentlich [44].

Die hohe Dunkelziffer an asymptomatischen Infektionen unter wohnungslosen Menschen könnte eine Herausforderung für die versorgenden Systeme darstellen. Regelmäßige Tests auch bei asymptomatischen Besuchenden einer Unterkunft und verstärkte Testfrequenz, wenn vereinzelte Tests in Unterkünften positiv sind, könnten einem Ausbruchsgeschehen entgegenwirken.

Einen wichtigen Infektionsschutz ermöglicht außerdem eine Impfung gegen SARS-CoV‑2, die mit dem Jahresbeginn 2021 auch für wohnungslose Menschen zunehmend angeboten werden konnte. Ein bestehender Impfschutz gegen SARS-CoV‑2 konnte im Sommer 2021 laborchemisch bei 42 % der NAPSHI-Studienteilnehmenden nachgewiesen werden. Zum vergleichbaren Zeitpunkt waren in der deutschen Allgemeinbevölkerung 63,5 % mindestens einmal und 57,8 % der Menschen vollständig geimpft [49]. Eine Studie aus Nordrhein-Westfalen aus 2022 fasst die Situation bei wohnungslosen Personen zusammen: niedrige Impfquote, hohe Prävalenz [50].

Eine amerikanische Studie zeigte, dass das Aufsuchen von Einrichtungen der Wohnungsnotfallhilfe positiv mit einem bestehenden Impfschutz für SARS-CoV‑2 assoziiert war [17]. Die BAG W empfiehlt, niederschwellige Impfangebote für solche Menschen zu machen, die angeben, bisher nicht geimpft worden zu sein. Um dies zu erreichen, könnten Impfzentren unmittelbar in oder neben Wohnungslosenunterkünften eingerichtet werden. Zusätzlich ermöglicht der Einsatz von Impfmobilen oder Straßenambulanzen einen besseren Zugang zu Menschen, die das Hilfesystem sonst meiden [18]. Darüber hinaus ist für Hepatitis B beschrieben, dass regelmäßige Impfangebote in Gefängnissen, aber auch Substitutionseinrichtungen die Impfquote positiv beeinflussen könnten [51]. Gegebenenfalls sind solche Ansätze auch auf SARS-CoV‑2-Impfstrategien bei wohnungslosen Menschen zu übertragen.

Um effektiven Infektionsschutz bei wohnungslosen Menschen zu erreichen, muss die Heterogenität der Kohorte beachtet und Informationsstrategien angepasst werden. Dazu sollten versorgende Einrichtungen vernetzter zusammenarbeiten und mittels einfacher Sprache, gegebenenfalls mit Bildmaterial, über Infektionserkrankungen und deren Prävention aufklären und so zu einer höheren Impfbereitschaft beitragen [52, 53].

## Fazit

Im März 2020 machten die Autoren Tsai und Wilson mit als Erste auf das möglicherweise problematische Infektionsgeschehen unter wohnungslosen Menschen aufmerksam. Sie postulierten ein erhöhtes Risiko für COVID-19 unter wohnungslosen Menschen begründet durch ihre Umwelt- und Lebensbedingungen, wie zum Beispiel die Nutzung von Gruppenunterkünften, den erschwerten Zugang zu medizinischer Versorgung und Informationen sowie die eingeschränkten Möglichkeiten für notwendige Hygienemaßnahmen [1].

Mit Blick auf die bisher veröffentlichten Daten ist von einer hohen Vulnerabilität wohnungsloser Menschen während der COVID-19-Pandemie auszugehen: Zu Beginn der Pandemie mussten viele Versorgungseinrichtungen schließen oder ihre Angebote reduzieren, da Präventionsmaßnahmen vielfach nicht ausreichend gewährleistet werden konnten [10]. Eine fehlende Krankenversicherung erschwert für etwa 1/3 der wohnungslosen Menschen in Deutschland den Zugang zu ambulanten und stationären medizinischen Hilfsangeboten [5].

Darüber hinaus wurde vermutet, dass wohnungslose Menschen aufgrund hoher Prävalenzen psychischer und somatischer Erkrankungen ein erhöhtes Risiko für einen schweren Verlauf einer SARS-CoV‑2-Infektion hätten. Studien bestätigen hohe Prävalenzen psychischer und somatischer Erkrankungen unter wohnungslosen Menschen in Deutschland [5, 13, 22]. Dennoch konnte die Hypothese besonders schwerer COVID-19-Verläufe unter wohnungslosen Menschen nicht belegt werden. Im Gegenteil ergab sich ein relevant erhöhter Anteil an asymptomatisch Infizierten gegenüber der Allgemeinbevölkerung [42, 46].

Wie vermutet, zeigten sich im Verlauf der COVID-19-Pandemie bei wohnungslosen Menschen häufiger als in der Allgemeinbevölkerung Hinweise auf eine SARS-CoV‑2-Infektion [39, 41, 42]. Besonders die hohe Rate an unwissentlich infizierten Personen könnte zur Verbreitung der Viruserkrankung beigetragen haben [47, 48]. Trotzdem konnte ein sozialmedizinischer Eklat unkontrollierter COVID-19-Ausbruchsgeschehen durch wohnungslose Menschen, vor dem Tsai und Wilson zu Beginn der Pandemie warnten, nicht beobachtet werden.

Einen wesentlichen Teil zu dieser positiven Entwicklung könnten Einrichtungen der Wohnungslosenhilfe in Deutschland beigetragen haben. Zwar zeigten sich erhöhte Infektionsraten unter wohnungslosen Menschen, die in Unterkünften übernachten, jedoch leisteten die Versorgenden neben akuter medizinischer Hilfe auch Aufklärung über Infektionserkrankungen, konnten niederschwellig Test- und Impfangebote bereitstellen und eine Anlaufstelle für Menschen sein, die sonst nur schwer Zugang zum Regelsystem finden.
